# Genomic Diversity of Torque Teno Virus in Blood Samples from Febrile Paediatric Outpatients in Tanzania: A Descriptive Cohort Study

**DOI:** 10.3390/v14081612

**Published:** 2022-07-23

**Authors:** Florian Laubscher, Mary-Anne Hartley, Laurent Kaiser, Samuel Cordey

**Affiliations:** 1 Laboratory of Virology, Department of Diagnostics, Geneva University Hospitals & Faculty of Medicine, University of Geneva, 1205 Geneva, Switzerland; florian.laubscher@hcuge.ch (F.L.); laurent.kaiser@hcuge.ch (L.K.); 2 Intelligent Global Health Research Group, Machine Learning and Optimization Laboratory, Swiss Institute of Technology (EPFL), 1015 Lausanne, Switzerland; mary-anne.hartley@epfl.ch; 3 Division of Infectious Diseases, Geneva University Hospitals, 1205 Geneva, Switzerland; 4 Geneva Centre for Emerging Viral Diseases, Geneva University Hospitals, 1205 Geneva, Switzerland

**Keywords:** torque teno virus, genomic diversity, children

## Abstract

Torque teno virus (TTV) is considered to be an ubiquitous member of the commensal human blood virome commonly reported in mixed genotype co-infections. This study investigates the genomic diversity of TTV in blood samples from 816 febrile Tanzanian children. Metagenomic next-generation sequencing was used to screen for TTV in individual blood samples from a cohort of 816 febrile Tanzanian paediatric outpatients. For positive samples, the number of TTV species and genotypes present were evaluated. We investigate the linear relationship between individual TTV diversity and the patient age by linear regression. TTV was detected in 97.2% of sera. ORF1 analysis revealed the presence of 149 genotypes from 38 species, suggesting the presence of 13 new species. These genotypes were mostly present as co-infections with a median of 11 genotypes/subject (range: 1–71). In terms of species, we found a median of nine species/subject (range: 1–29). We further show a significant association between the diversity of co-detected TTV and the age of the subjects (*p* value < 0.0001). This study shows that significant TTV genomic diversity is acquired by the age of five and that this diversity tends to increase with age, which indicates a repetitive TTV acquisition during the first months/years of life.

## 1. Introduction

Torque teno virus (TTV) belongs to Alphatorquevirus genus, one of the 30 genera of the *Anelloviridae* family according to the recent taxonomic update of mammalian anelloviruses [1]. Using a 69% sequence identity threshold in the ORF1 coding region, 22 TTV species have been defined by the *Anelloviridae* Study Group of the International Committee on Taxonomy of Viruses (ICTV). TTV are non-enveloped viruses, containing a circular, negative single-stranded DNA genome of 3.6 to 3.9 kb in length. Although first described more than 20 years ago in the serum of a Japanese patient with hepatitis of unknown aetiology [2], TTV did not have a specific causal link to disease. The modes of transmission are not yet elucidated and most likely multiple (e.g., faecal–oral, blood–blood, airway, vertical, sexual, etc. [3]) as it has been detected in various potentially contagious biological samples (e.g., blood, saliva, urine, faeces, nasal secretions, semen, breast milk, etc.) [4,5,6]. While its presence in various organs and tissues suggests that TTV does not have a specific tropism, peripheral blood mononuclear cells and lymphocytes are suggested as one of its main replication-competent cells [7,8], with more than 1 × 10^10^ virions produced daily [9].

TTV is ubiquitous in humans, with an estimated prevalence over 50% in the healthy general population [6]. It is thought that acquisition occurs during the first months/years of life [10,11,12], where blood prevalence is detected in infants and increases with age in the first months of life, suggesting perinatal transmission [11,12,13,14,15]. The current consensus is that TTV are non-pathogenic and are thus considered as commensals of the blood virome. Furthermore, several studies recently investigated the potential role of TTV as a biomarker of immunity, suggesting that the viral load could predict post-transplant clinical outcomes [3]. Next-generation sequencing (NGS) technologies have made it possible to investigate intra-host genomic diversity at an unprecedented level of granularity. It is now established that humans are very frequently co-infected by several species and genotypes [16,17,18], with the possible detection of a considerable number of genotypes within a single individual and that this diversity may remain stable over time [19]. The reason for this genetic diversity is not yet clear. In contrast to its conserved untranslated region, coding regions (especially ORF1) show the highest sequence heterogeneity. TTV is a DNA virus, making it unlikely (but not impossible) to have a high mutation rate. Interestingly, several studies have shown recombination as a key driver of the TTV genetic diversity [16,20,21,22].

We previously described the blood virome (sera) in a large cohort of febrile Tanzanian children (<5 years) by metagenomics NGS (mNGS) and noted the high prevalence and genetic heterogeneity for TTV. Here, we use raw data generated in the context of our previous mNGS study within this paediatric population [23] to carry out an in-depth analysis of the TTV genomic diversity at the species and genotype levels using the new classification criteria established by the ICTV, as well as the new publicly available full genome TTV sequences. Furthermore, we investigate the relationship between TTV diversity as a function of patient’s age.

## 2. Materials and Methods

### 2.1. Origin of mNGS Raw Data Used in This Study

**Figure 1:**
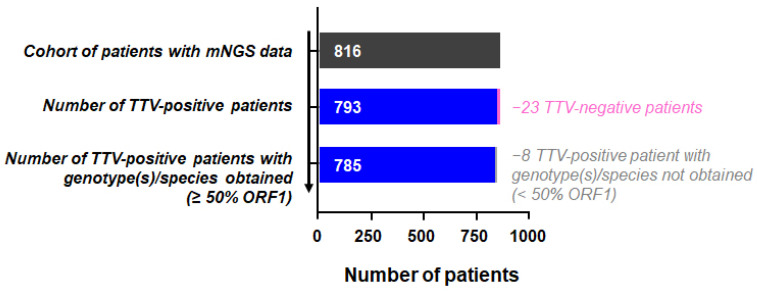
Flowchart of study and TTV analysis.

Raw data were generated in the context of a previous study (registered in ClinicalTrials.gov, identifier NCT02225769) that aimed to characterize the blood virome (sera) in a cohort of 816 children (2 to 59 months of age, median = 14.77) consulting at outpatient departments in Dar es Salaam, Tanzania, for acute fever (Figure 1; Table S1) [23]. Patients were recruited between December 2014 and February 2016 and diagnosed according to a standardized clinical decision support algorithm [24]. The 816 patients used in this study were selected from a larger cohort of 3192 patients to enrich the diagnoses of (1) “Fever without focus” (n = 638), (2) “Severe illness” (n = 101) and (3) “Malaria” (n = 123). Some patients may belong to more than one group.

### 2.2. Bioinformatic Analysis

Here, we analysed in detail the TTV reads previously generated using the “DNA protocol” [23] from libraries prepared with Illumina Nextera XT (12 PCR cycles) (Illumina, San Diego, CA, USA), then multiplexed by six on the HiSeq 4000 platform (Illumina, 2 × 100 bp paired-end protocol).

#### 2.2.1. Database

In order to create a database of complete Alphatorquevirus ORF1 sequences annotated at species and genotype levels for metagenomic analysis, we updated the TTV part of our former in-house *Anelloviridae* database. Our former database was composed of representative (90% identity) complete ORF1 sequences, and the sequences were either downloaded from GenBank (downloaded March 2019) or de novo assembled from our previous study (GenBank submitted: MN765195 to MN768170) [23].

The database was updated by adding new complete ORF1 TTV sequences from GenBank using organism query “alphatorquevirus” and “unclassified *Anelloviridae*” at the date of 16 November 2021.

In addition, we added all sequences listed as “Alphatorquevirus” in the last taxonomic update of the mammalian anelloviruses Supplementary Table (Taxonomic update for mammalian anelloviruses (family *Anelloviridae*)). Duplicated sequences were removed and completed ORF1s were inferred from downloaded sequences using GenBank annotations, when possible, and if not, using an ORF detection script. “ACG” was also used as an alternative initiation codon for ORF1 detection in the TTV of the phylogenetic group 4. Sequences were then checked to belong to Alphatorquevirus genus and merged with our former database.

In order to annotate sequences at a species level, we used the ICTV threshold of 69% nucleotide identity on complete ORF1 (Taxonomic update for mammalian anelloviruses (family *Anelloviridae*)) [1]. The species demarcation threshold was checked in a two-by-two sequence alignment using MUSCLE (v3.8.31). Sequences were regrouped together if reach the threshold, checking that groups that had two sequences that reach the threshold were merged. Existing species names were tagged to each group. Groups not falling into existing species were classified as new Torque teno species names and numbered from 35 to avoid confusion with names already existing in GenBank (e.g., with Simian torque teno virus 34 (Torque teno chlorocebus virus 2)). Using cdhit-est (CD-HIT v4.7), we ended up with a new database of 90% identity representative sequences.

To annotate sequences at the genotype level, we created phylogenetic trees, where nucleotide sequence sets with 90% ORF1 identity were subdivided in 6 phylogenetic groups (1, 2, 4, 5, 6 and 7) and 4 subgroups (3, 3a, 3b and 3c). For each, a multiple alignment was performed using MUSCLE (v3.8.31). General Time Reversible model with a discrete Gamma distribution and invariable site allowed (GTR+G+I) was the best fitting nucleotide evolutionary model for the data using MEGA X (v10.0.5). A tree was then generated using the Maximum Likelihood method and GTR+G+I model.

Genotypes were assigned using a threshold of 80% nucleotide identity on complete ORF1 sequences (based on a two-by-two sequence alignment within species). For each genotype, a letter has been assigned according to the phylogenetic group/subgroup 1, 2, 3, 3a, 3b, 3c, 4, 5, 6 and 7, respectively “A”, “B”, “C”, “D”, “E”, “F”, “G”, “H”, “J” and “K” (“I” was skipped to avoid possible confusion with lowercase letter “l”) and numbered starting from 1. The monophyletic character of each genotype was checked using the group/subgroup trees. Genotypes were then propagated to other sequences of the 90% identity clusters to obtain a list of complete ORF1 sequences of each genotype (Table S2).

#### 2.2.2. Mapping

DNA FASTQ files were filtered to remove adapters and low-quality sequences using Trimmomatic (v0.36). Human reads were then filtered out by mapping to the human genome and transcriptome (hg38, gencode.V23) using snap-aligner (v1.0beta.18). Low complexity reads were filtered using Tagdust (v2.31). Filtered reads were then mapped to the TTV database using snap-aligner (v1.0beta.18). Total matching reads and genome coverage were computed for each genome detected and results with the best genome coverage for each genotype were reported. A cross-contamination threshold of 1% reads was applied to avoid false positives in the same sequencing lane. Detection of a genotype/species was reported if ≥50% of the ORF1 was covered.

### 2.3. Circos Plot

A Circos plot was generated using R scripts (v3.4.4) with ggplot2 (v0.3.3), ggraph (v2.0.5) and igraph (v1.2.11) libraries. For each genotype, numbers of co-infections with each other genotype were computed, co-infections links were shown for rate ≥ 75%. A genotypes/species hierarchical structure dataframe was used to determine node positions of the links (intra- or inter-species). Vertices dots were positioned by phylogenetic groups and seized by number of detections.

### 2.4. Statistical Analyses

Statistical analyses (simple linear regressions) were performed using GraphPad Prism 9.0.2 (GraphPad Software, Inc., San Diego, CA, USA).

## 3. Results

**Figure 2:**
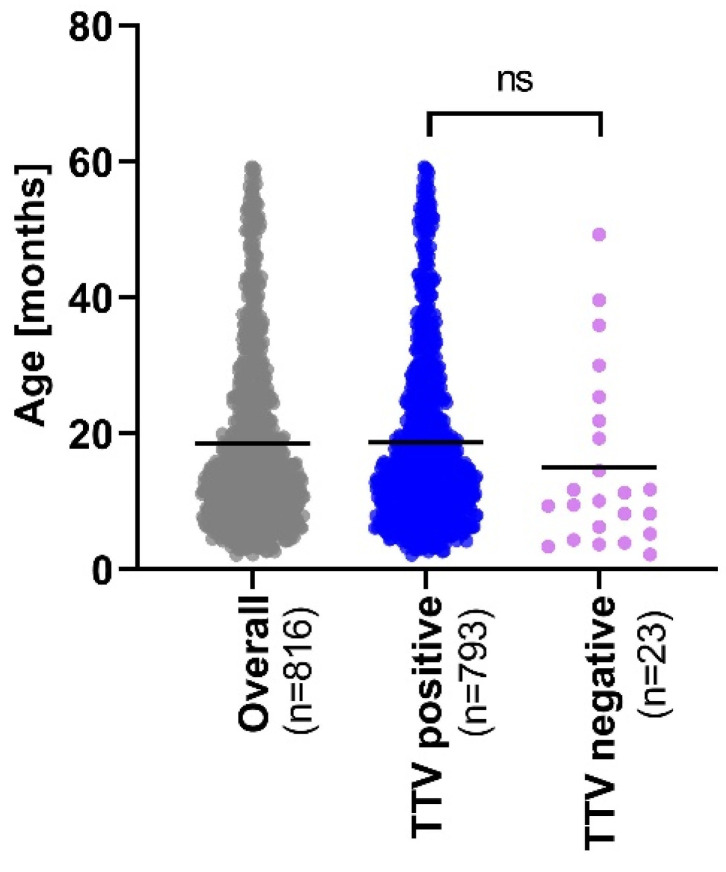
Age repartition. Age repartition of the 816 patients enrolled in this mNGS study. Medians are reported by black bars. Unpaired t-test was used to compare means. ns, nonsignificant.

Of the 816 sera (from 816 individual paediatric subjects), TTV specific reads are detected in 793 samples (97.2%) (Figure 1). A mean of 5.46 × 10^7^ paired reads is obtained across all samples (range: 2 × 10^6^–2.52 × 10^8^) with a median of 6.75 × 10^4^ specific TTV paired reads per positive sample (range: 7–1.63 × 10^7^, Figure S1). Since the total number of paired reads obtained by mNGS is higher in TTV-negative than TTV-positive patients (mean = 5.87 × 10^7^ and 5.44 × 10^7^, respectively; *p* value = 0.0272; Table S1), the risk of false TTV negatives is extremely limited. We observe no correlation between the number of TTV-specific reads in clinical samples and the age of the subjects (*p* value = 0.3033, R2 = 0.001340) (Figure S2). Patient age is also not significantly different between TTV-positive and -negative paediatric subjects (Figure 2).

### 3.1. TTV Genomic Diversity

**Figure 3:**
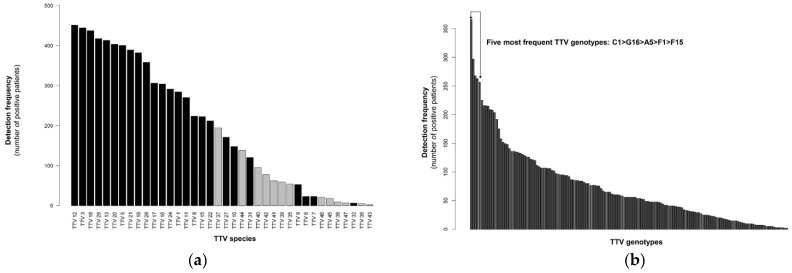
Frequency of each TTV species and genotype. (**a**) Barplot showing the number of positive patients for each TTV species detected by mNGS. The 25 TTV species known to infect humans as well as the 13 additional species reported in this study are represented by black and grey bars, respectively; (**b**) Barplot showing the number of positive patients for each TTV genotype detected by mNGS.

For each TTV-positive sample, a genotype/species assessment is systematically performed (when ≥50% the ORF1 nucleotide sequence was available). A phylogenetic analysis based on the complete ORF1 nucleotide sequence reveals the presence of 38 species (Figure 3a) spread out into 10 phylogenetic groups/subgroups (Figure S3). Among these 38 species, 25 are known to infect humans, suggesting the presence of 13 additional species (named TTV 35 to TTV 47 to avoid any confusion with Torque Teno Chlorocebusvirus (TTCV) indicated as TTV 33 and 34 on GenBank). The latter are less frequently detected in this cohort than the 25 species known to infect humans (Figure 3a).

In all, 149 of the 151 assigned human TTV genotypes in our database are detected in this paediatric cohort. The two undetected genotypes are B17 (GenBank accession number AB054648) and J3 (GenBank accession number KT163896) from TTV 8 and 32 species, respectively. The most frequent TTV genotypes observed are C1 (n = 364), G16 (n = 298), A5 (n = 268), F1 (n = 263) and F15 (n = 257) (Figure 3b). No single TTV genotype is detected in all of the TTV-positive patients, reflecting the absence of a common TTV background in this paediatric cohort. Each TTV genotype detected by mNGS in this study is reported in at least two subjects (Figure 3b).

### 3.2. TTV Species and Genotype Co-Detections

**Figure 4:**
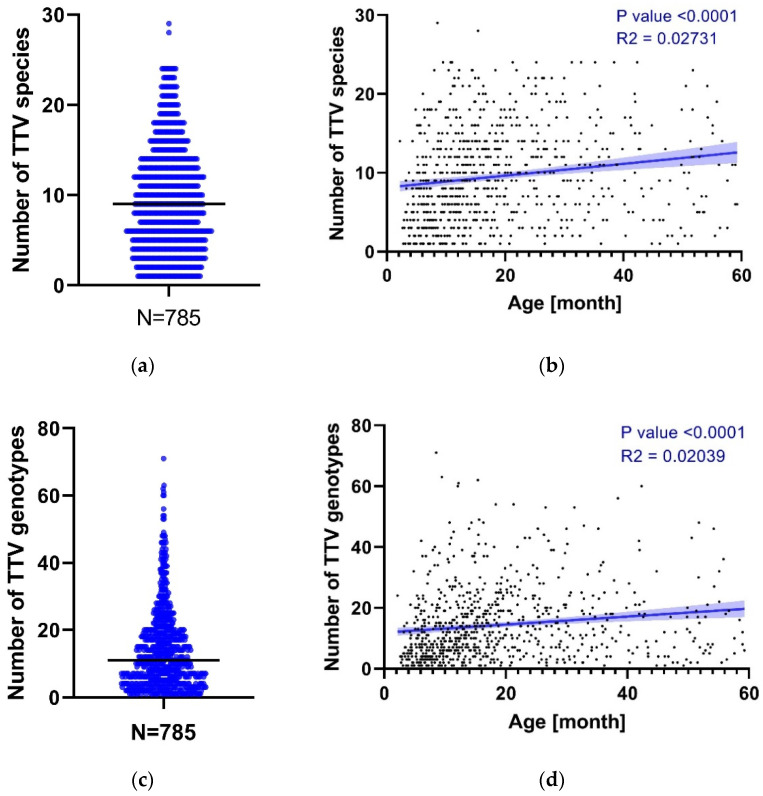
TTV genomic diversity in serum. (**a**) Number of TTV species detected by mNGS for each patient. Median is reported (black bar); (**b**) Simple linear regression analysis of the number of TTV species and the age of the patients (in months). Each black dot represents one of the 785 positive patients; (**c**) Number of TTV genotypes detected by mNGS for each patient. Median is reported (black bar); (**d**) Simple linear regression analysis of the number of TTV genotypes and the age of the patients (month). Each black dot represents one of the 785 positive patients for which a genotype could be obtained (≥50% ORF1).

Of the 793 TTV-positive sera, 8 genotypes/species could not be reliably obtained (<50% ORF1) (Figure 1). The remaining 785 have a median of 9 species per sample (range: 1–29) (Figure 4a), with at least 2 different TTV species detected in 743/785 (94.6%). Using simple linear regression to assess the association of species diversity with age, we find a significant positive correlation for the number of TTV species detected in sera as a function of age (*p* value < 0.0001, R^2^ = 0.02731) (Figure 4b). A similar analysis at the genotype level reveals a median of 11 genotypes per sample (range: 1–71), where 746/785 samples (95%) are “co-infected” by different TTV genotypes (Figure 4c). For these 785 sera, the number of reads obtained for each TTV genotype detected per sample is reported in Table S3. The analysis of the distribution of the number of reads for each TTV genotype (n = 149) reported in this cohort reveals a large disparity for each genotype with no evidence for any of them to be associated with a higher or lower number of reads (Figure S4). It is noteworthy that, although an average of 5.46 × 10^7^ paired reads were obtained across all samples (which is consequent for viral mNGS investigations), we cannot rule out that, given the high number of co-detected TTV genotypes per sample, some with a low viral load were not detected in some samples. Simple linear regression on age shows a significant trend towards an increase in the number of TTV genotypes detected in sera as a function of age (*p* value < 0.0001; R^2^ = 0.02039) (Figure 4d). An age distribution of TTV genotypes analysis does not reveal any earlier acquired genotypes (Figure S5).

### 3.3. TTV Genotypes Relationship Analysis

**Figure 5:**
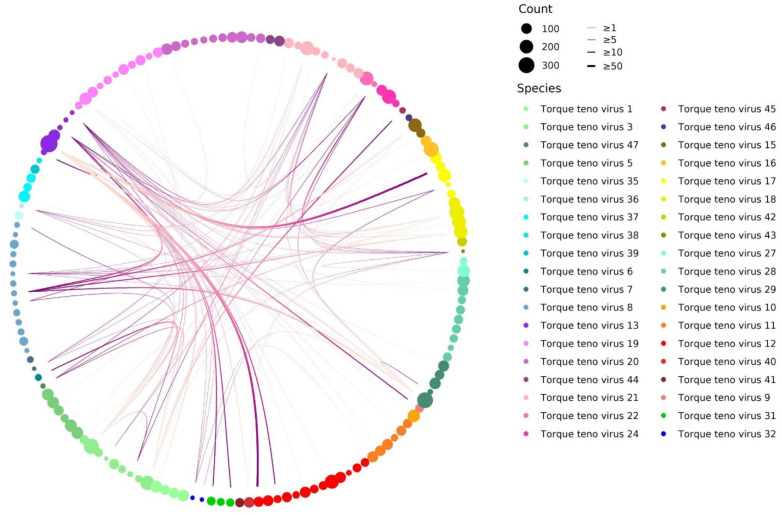
Relationship between co-detected TTV genotypes within a same patient. Each of the 149 TTV genotypes reported by mNGS in this paediatric cohort is represented by a dot of variable size depending on the number of times (i.e., counts) it is detected. Genotypes are grouped by species, and each of the 38 TTV species reported in this study being represented by a specific colour code. Each line indicates which TTV genotype (dark purple end) is frequently co-detected with which other (light purple end). A co-detection cut-off of ≥75% is used in order to illustrate only strong TTV genotypes co-detection rates.

Given that 95% of subjects are co-infected by different TTV genotypes, we assess the presence of co-dependences of specific genotype(s). In order to highlight only strong TTV genotypes co-detection rates, we set a co-detection cut-off ≥75% (Figure 5). The Circos plot shows for 27 of the 149 TTV genotypes a total of 100 strong co-detections of one genotype to another, and none are reciprocal. They are, however, mostly observed for genotypes infrequently detected in this study and infrequently concern (5%) one genotype with another from the same species (Figure 5). No TTV genotype necessarily depends (i.e., co-detection rate 100%) on the presence of another.

The relative abundance analysis of TTV genotypes shows a low median (<10%) in the vast majority of them (Figure S6), reflecting the large number of genotypes co-detected per sample. Although most genotypes may occasionally become the majority in some samples, the eight with a median >25% are rarely detected in this paediatric cohort (maximum 24× for genotype B12). It is interesting to note that six of the eight belong to the TTV 8 species (genotypes B12, B13, B14, B18, B21 and B23).

## 4. Discussion

Here, in this cohort of febrile Tanzanian children, we not only report a significant TTV genetic diversity within individual patients but also a significant trend toward an increase in the TTV diversity (genotypes and species) as a function of the age. Taken together, the high prevalence of TTV (97.2%, 793/816) in this young cohort and the significant trend of age-dependent genotypic diversity, indicates perinatal acquisitions with repetitive early exposure during the first months/years of life. Although some TTV genotypes are more frequently detected in this paediatric cohort, our results speak in favour of a distinctive rather than a common “TTVome”. Furthermore, this study reveals neither earlier acquisition of specific TTV genotypes nor any reciprocal co-dependences between two or more TTV genotypes, which also indicates genotype independence where co-infections are more likely the result from random extrinsic acquisition rather than intrinsic mutation. It is noteworthy that, while an in-depth analysis of the clinical factors associated with TTV prevalence is out of the scope of this descriptive analysis, we found no correlation to the three diagnostic subgroups selected in this cohort (i.e., malaria, severe disease, or fever without source).

Some longitudinal studies have previously investigated the dynamics of TTV during the first years of life by comparing the prevalence as well as the evolution of viral load over time [11,12,13]. In a study including 22 mother–child blood pairs with a 30-month follow-up period for children, Komatsu et al. reported an increase in the prevalence of TTV infections with age, with a total of 13 children that became TTV-positive by PCR during their follow-up period [14]. Remarkably, the authors observed a same predominant TTV genotype between the child and the mother at the first PCR-positive time point in 11/13 pairs (85%), suggesting that mothers represent the main source of infection to child, while thereafter the predominant genotype changed in half of the children detected positive (5/10) at ≥two time-points during the follow-up period, suggesting other environmental sources of infections. However, these authors used PCR-amplicon cloning methods followed by classical sequencing of 10 clones by individuals, thereby limiting the depth of analysis compared to the NGS method performed in this study.

Anelloviruses are known to have significant genetic diversity. The reasons for this are not fully understood and could result from a high mutation rate (although this is not typical of DNA viruses), to reflect millions of years of evolution from a common ancestor in a manner similar to what has been suggested for papillomaviruses [25], or may also be due to recombination events which seem to be the key driver of annellovirus evolution. With a total of 149 TTV genotypes detected, as well as 13 potential additional species, our study suggests that it is likely that much more remain to be discovered.

This study has some limitations. We complied with the TTV classification criteria used by the ICTV, (i.e., analysis of ORF1 with a 69% sequence identity threshold). Therefore, we cannot exclude missing some recombination events. The results reported here were obtained from a cohort of febrile children and need to be reproduced in a cohort of asymptomatic children in order to draw definitive conclusions. Finally, sera were not spiked individually with a standardized internal positive control. Indeed, in the initial study, “no-template” and positive controls were included in each sequencing run to assess the presence of potential mNGS contaminants and the entire process efficiency, respectively. As a result, a normalization of the number of reads obtained for each TTV genotype detected per sample was not considered. Finally, we cannot rule out that given the high number of co-detected TTV genotypes per sample or the presence of other DNA viruses recognized or not of clinical significance which could compete for sequencing that some TTV genotypes with low viral load were not detected in some samples.

Our study observes an increase in TTV genetic diversity with age in a cohort of febrile Tanzanian children under 5 years. Our study raises new questions, such as whether this trend continues during the rest of childhood and how long the diversity is maintained. It would be interesting to assess if there is a preferential selection of certain genotypes within an individual, and if so when and by which mechanism? As sera were collected in a cross-sectional manner (at the time of the paediatric emergency room visit), longitudinal analysis of TTV diversity within individuals was not possible.

## 5. Conclusions

In conclusion, our investigations highlights the need for further large-scale longitudinal investigations of TTV genetic diversity in symptomatic and asymptomatic children from various parts of the world.

## Data Availability

All scripts used can be found at https://github.com/Laubscher/Anelloviruses (release date 3 June 2022). Sequencing data: Bioproject PRJNA666535.

## Supplementary Materials

The following supporting information can be downloaded at: https://www.mdpi.com/article/10.3390/v14081612/s1, Figure S1: Number of TTV species detected by mNGS for each patient; Figure S2: Simple linear regression analysis of the number of TTV reads and the age of the patients (month); Figure S3: Maximum likelihood phylogenetic tree of TTV using the ORF1 nucleotide sequences; Figure S4: Distribution of the number of reads for each TTV genotype; Figure S5: Age distribution of TTV genotypes; Figure S6: Relative abundances of TTV genotypes; Table S1: patient’s dataset; Table S2: Database complete Alphatorquevirus genotypes; Table S3: Number of reads detected for each TTV genotype per sample.
