# Supplementary material for: Genomic Diversity of Torque Teno Virus in Blood Samples from Febrile Paediatric Outpatients in Tanzania: A Descriptive Cohort Study
Source: Viruses. 2022 Jul 23;14(8):1612. doi: 10.3390/v14081612 (PMC9330782; doi:10.3390/v14081612)
Supplement: Supplementary file 1 [file viruses-14-01612-s001.zip › viruses-1778013-supplementary/Supplementary Figures and Tables legends.pdf]

Figure S1: Number of TTV species detected by mNGS for each patient. Median is reported (black bar).

Figure S2: Linear regression analysis of the number of TTV reads and the age of the patients (month). Each black dot represents one TTV positive patient.

Figure S3: Maximum likelihood phylogenetic tree of TTV using the ORF1 nucleotide sequences. Each phylogenetic group is shown individually. Non-human primate TTV genotypes/species are specifically notified.

Figure S4: Distribution of the number of reads for each TTV genotype. Median is reported (black bar).

Figure S5: Age distribution of TTV genotypes. Median is reported (black bar).

Figure S6: Relative abundances of TTV genotypes. Median is reported (black bar).

Table S1: **patient's dataset**

Table S2: **Database complete Alphatorquevirus genotypes.** For each TTV genotype in our database the corresponding GenBank accession number is provided.

Table S3: **Number of reads detected for each TTV genotype per sample.**
