# Supplementary figures and images for: Genomic Diversity of Torque Teno Virus in Blood Samples from Febrile Paediatric Outpatients in Tanzania: A Descriptive Cohort Study
Source: Viruses. 2022 Jul 23;14(8):1612. doi: 10.3390/v14081612 (PMC9330782; doi:10.3390/v14081612)

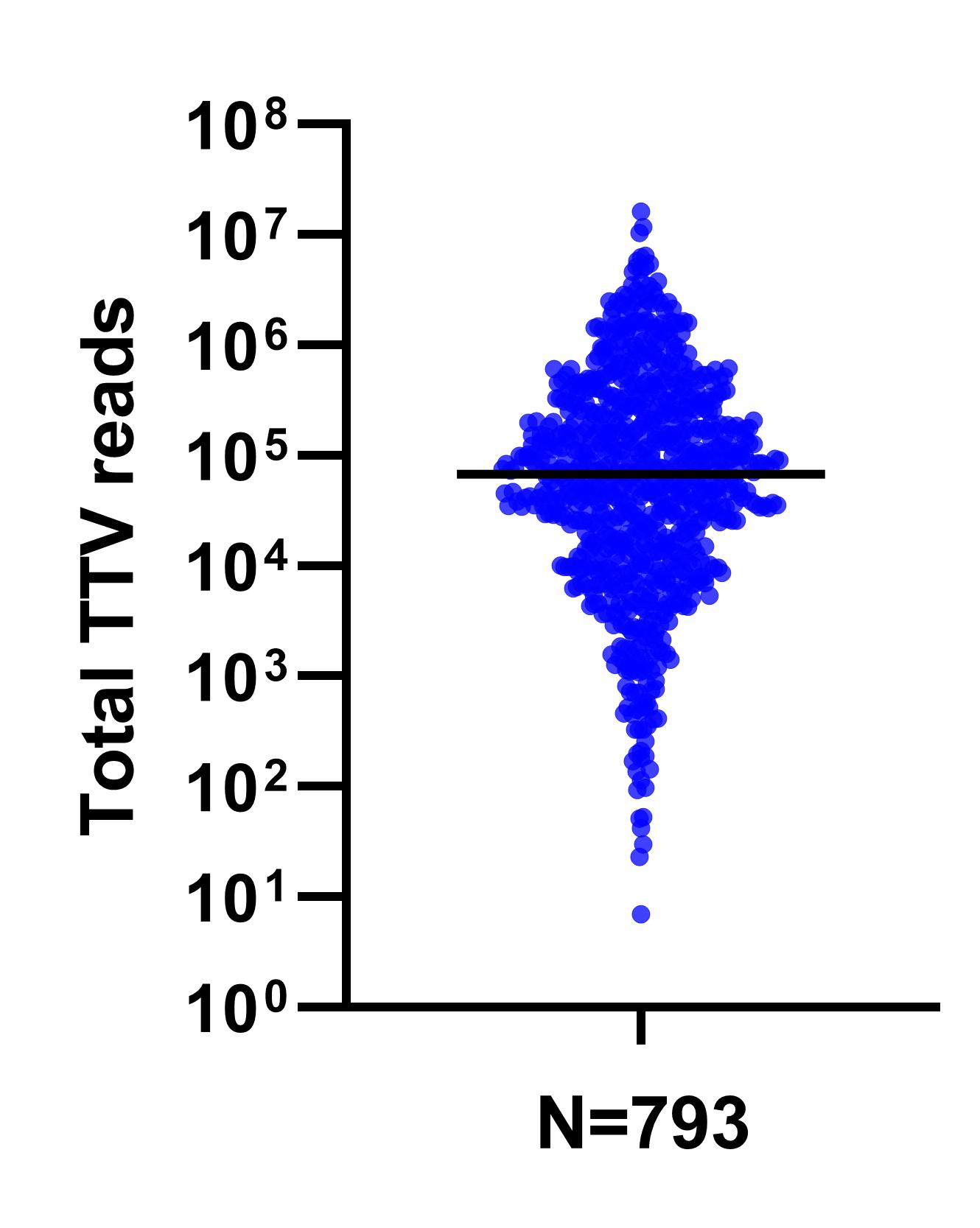

Supplement: Supplementary file 1 [file viruses-14-01612-s001.zip › viruses-1778013-supplementary/Figure S1.jpg]

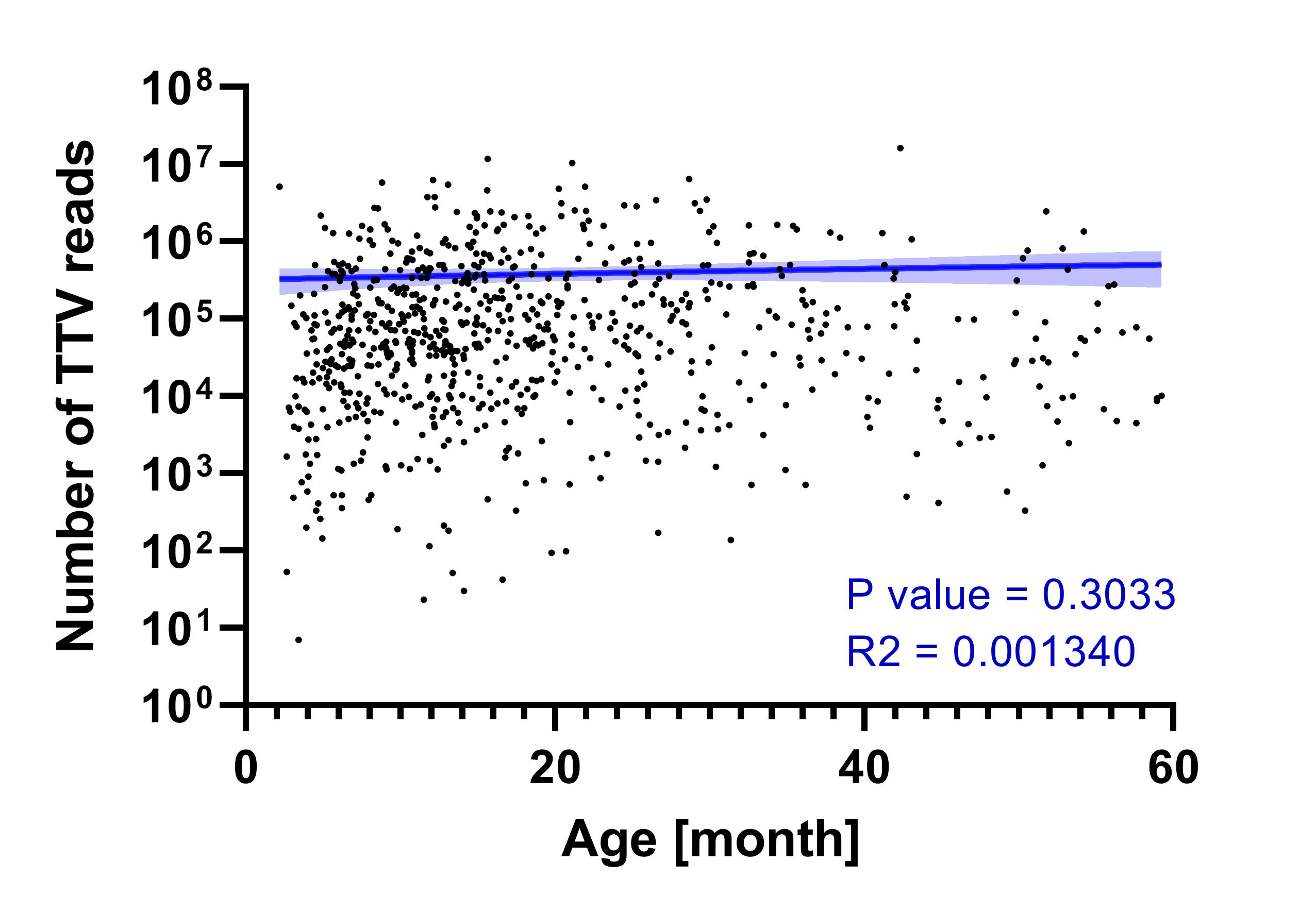

Supplement: Supplementary file 1 [file viruses-14-01612-s001.zip › viruses-1778013-supplementary/Figure S2.jpg]

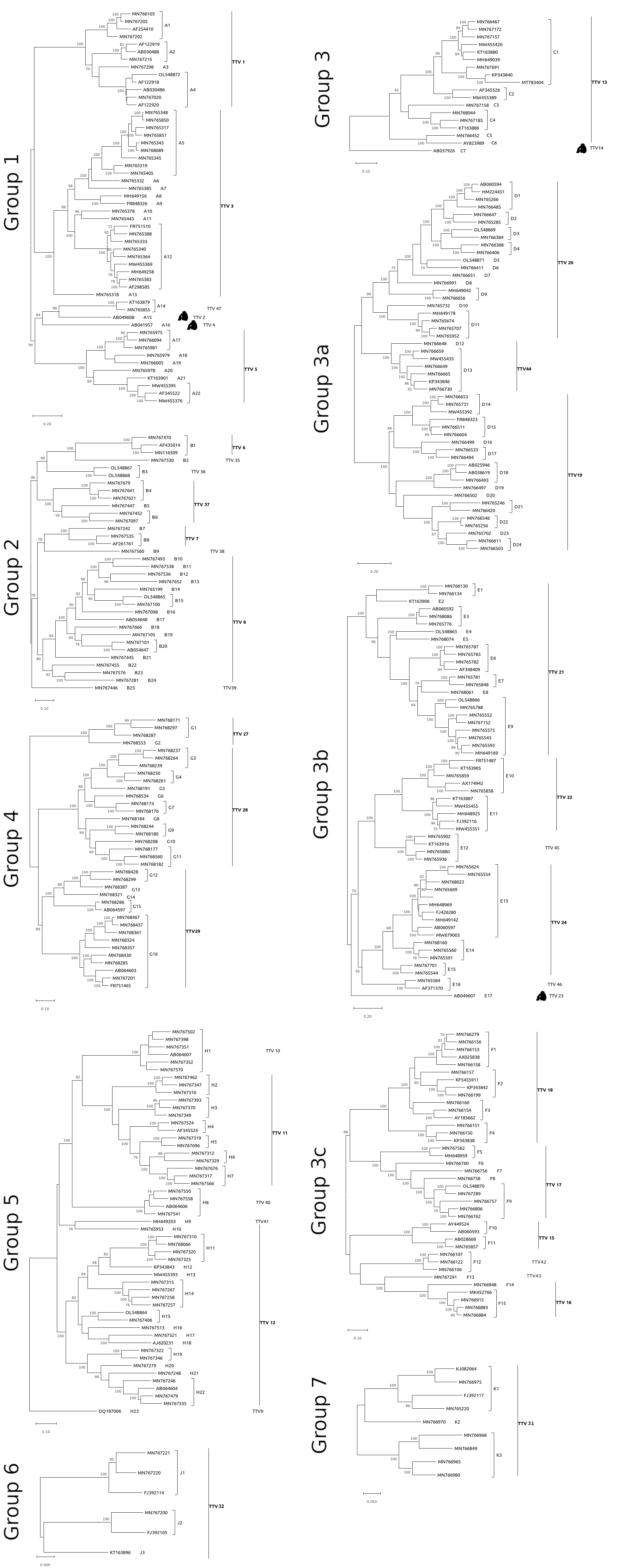

Supplement: Supplementary file 1 [file viruses-14-01612-s001.zip › viruses-1778013-supplementary/Figure S3.jpg]

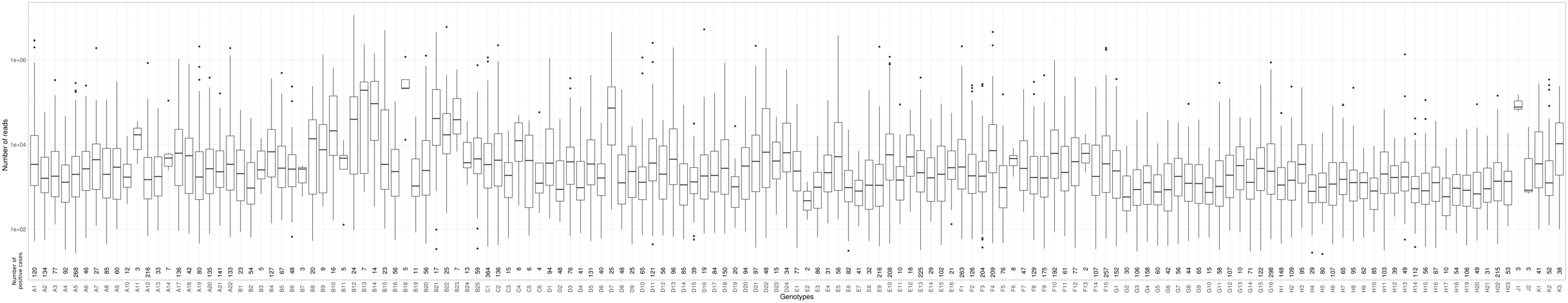

Supplement: Supplementary file 1 [file viruses-14-01612-s001.zip › viruses-1778013-supplementary/Figure S4.jpg]

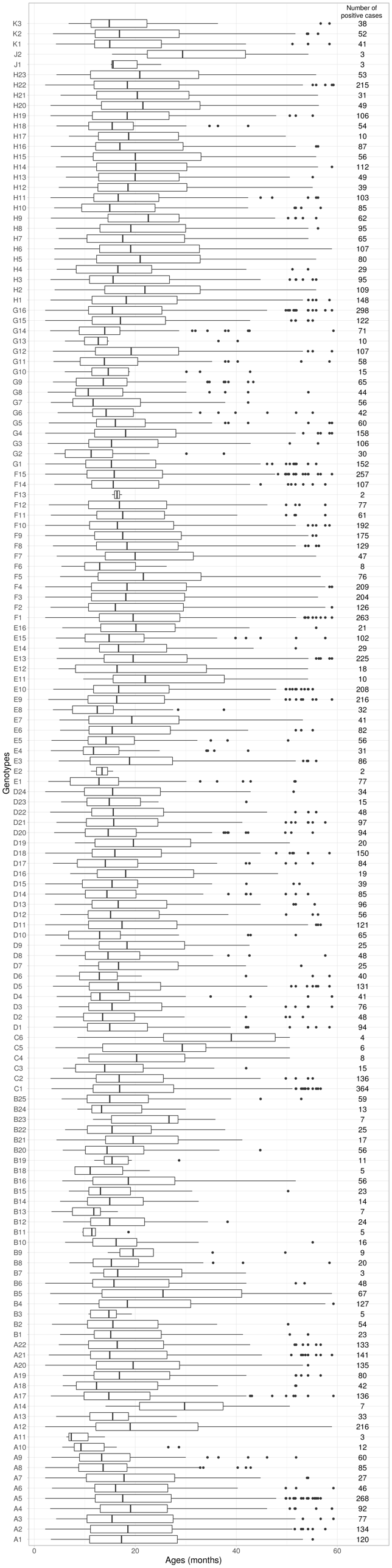

Supplement: Supplementary file 1 [file viruses-14-01612-s001.zip › viruses-1778013-supplementary/Figure S5.jpg]

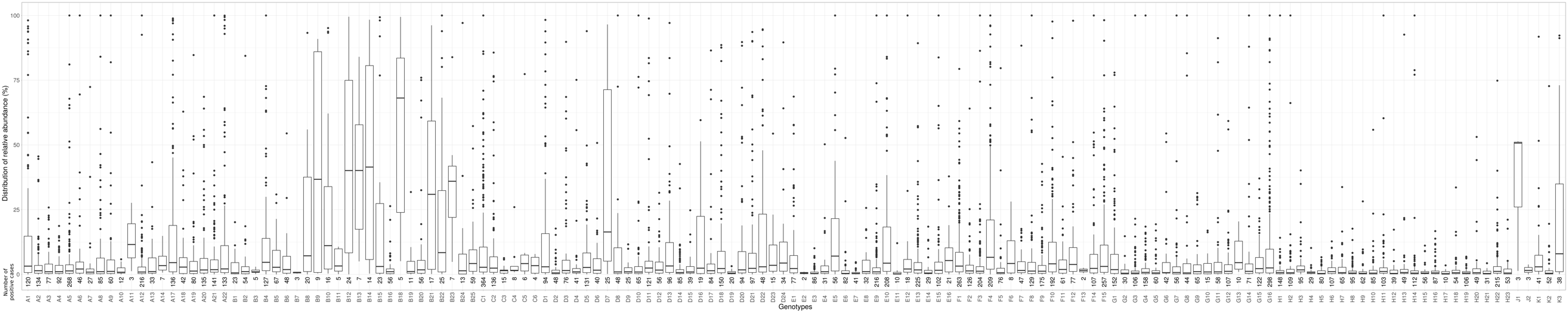

Supplement: Supplementary file 1 [file viruses-14-01612-s001.zip › viruses-1778013-supplementary/Figure S6.jpg]
